# Gender Differences in Long-Term Outcomes of Medical Therapy and Successful Percutaneous Coronary Intervention for Coronary Chronic Total Occlusions

**DOI:** 10.1155/2019/2017958

**Published:** 2019-09-10

**Authors:** Lei Guo, Haichen Lv, Lei Zhong, Jian Wu, Huaiyu Ding, Jiaying Xu, Rongchong Huang

**Affiliations:** Department of Cardiology, The First Affiliated Hospital of Dalian Medical University, Dalian City, China

## Abstract

**Background:**

There is a paucity of information about the gender differences in clinical outcomes of successful percutaneous coronary intervention (PCI) compared with medical therapy (MT) in patients with coronary chronic total occlusions (CTOs).

**Objectives:**

We aimed to investigate the impact of gender on long-term clinical outcomes associated with successful CTO-PCI versus MT in patients with CTOs.

**Methods:**

Between January 2007 and December 2016, a total of 1702 patients with ≥1 CTO were enrolled. After exclusion, 1294 patients with 1520 CTOs were analyzed and were divided into the female group (*n* = 304, 23.5%) and the male group (*n* = 990, 76.5%). The patients in the female or male group were assigned to a MT group or successful CTO-PCI group according to the treatment strategy. In the female group, they were divided into two groups: 177 patients in the MT group and 127 patients in the successful CTO-PCI group. In the male group, they were divided into two groups: 623 patients in the MT group and 367 patients in the successful CTO-PCI group. The primary outcome was cardiac death. The secondary outcome was major adverse cardiac event (MACE).

**Results:**

The median overall follow-up duration was 3.6 (IQR, 2.1–5.0) years, there were no significant differences between the MT and successful CTO-PCI groups with respect to the prevalence of cardiac death (MT vs. successful PCI: 6.8% vs. 3.9%, *p*=0.287) and MACE (20.9% vs. 21.3%, *p*=0.810) in female patients. In the male group, the occurrence of cardiac death (MT vs. successful PCI: 6.6% vs. 3.8%, *p*=0.066) was similar between the two groups. The MACE rate (30.0% vs. 18.5%, *p* < 0.001) was significantly higher in the MT group. Heart failure (hazard ratio 3.40, 95% confidence interval 1.23–9.40, *p*=0.018) was an independent predictor of cardiac death in female patients.

**Conclusions:**

Successful CTO-PCI was not associated with reduced risk of cardiac death compared with medical therapy alone in both female and male patients. However, men have a significant reduction in MACE rate after successful CTO-PCI. Aggressive CTO-PCI should be considered carefully among female patients.

## 1. Introduction

Gender differences have long been known to exist in the presentation and outcome of coronary artery disease (CAD). Multiple studies indicate that female patients are less likely than male to be referred for invasive coronary angiography and to undergo revascularization, despite almost the same prevalence of coronary disease [1, 2].

Chronic total occlusions (CTOs) represent an important and unique subgroup of coronary lesions and have been identified in up to 18% of all patients referred for diagnostic angiography [3, 4]. Most studies reported that successful CTO percutaneous coronary intervention (PCI) is associated with symptomatic relief of angina, improvement in left ventricular function, quality of life, and a reduction in mortality compared with failed CTO-PCI [5–7]. However, only approximately 10%–20.7% of CTOs are currently undergoing attempted CTO-PCI [3, 8], mainly because CTO-PCI procedures may be with relatively lower success rate, a higher risk of complication, and higher expense when compared with non-CTO elective PCI [9, 10]. Indeed, a substantial portion of CTO patients are treated with medical therapy (MT) alone instead of PCI [11, 12].

Female patients with CTOs have a greater incidence of comorbidities and a higher risk of intraoperative and postoperative complications compared with male patients [13–15]. Therefore, clinicians are more likely to treat these female patients who have CTOs with MT alone, and a previous study also reported female patients have the lowest rate of revascularization [16]. However, current CTO studies are typically comprised of less than 20% female patients, which is in accordance with the overall low inclusion of women in cardiovascular registries and randomized trials relevant to CTO [3, 13, 14], and there is relative paucity of information about the gender differences in clinical outcomes of successful CTO-PCI compared with MT for CTO patients. Moreover, most studies only focused on the outcomes between successful and failed CTO-PCI, the patients who undergo MT alone and did not undergo a CTO-PCI attempt were rarely considered previously [17]. Therefore, this study aimed to investigate the impact of gender on long-term clinical outcomes associated with successful CTO-PCI versus MT in patients with CTOs.

## 2. Methods

### 2.1. Study Population

**Figure 1:**
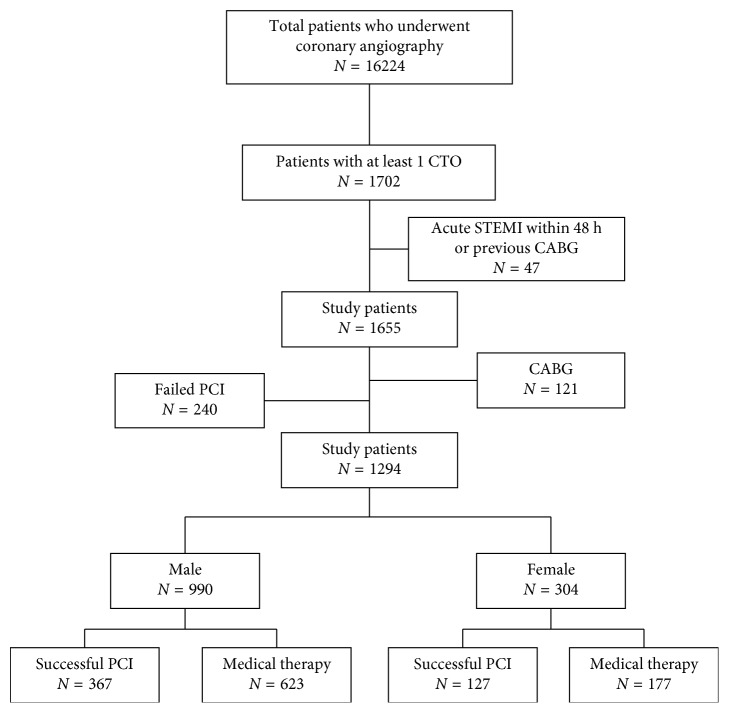
Flow chart of the study population. CABG, coronary artery bypass grafting; CTO, chronic total occlusion; PCI, percutaneous coronary intervention; STEMI, ST-segment elevation myocardial infarction.

The present study was a retrospective observational study. A total of 16224 patients who underwent diagnostic coronary angiography from January 2007 to December 2016 were included at the First Affiliated Hospital of Dalian Medical University (Dalian, China) [18]. Of these patients, 1702 had at least one CTO. 47 patients who underwent previous CABG and presented with acute myocardial infarction within 48 h were excluded. Among the 1655 patients, those who underwent CABG and failed CTO-PCI were excluded. Thus, 1294 patients with at least one CTO were included for analysis (Figure 1). Patients were grouped into the female group and the male group. The patients in the female group or male group were assigned to a MT group or successful CTO-PCI group according to the treatment strategy. Initial PCI or MT was selected according to the presence of symptoms, high comorbidity or high risk for revascularization, the suitability of the target distal vessel for revascularization (diameter > 2.5 mm), and patients' economic burden [18]. In asymptomatic patients who did not have viability data available or in subjects with proved absence of viability, MT was strongly preferred. In symptomatic patients, even without information on viability or in asymptomatic patients with viability, PCI was preferred. The decision to perform PCI for CTO patients was also dependent on several factors, including LV function, the extent of other coronary artery disease, CTO location, and technical difficulty. However, several other factors, including patient preference and their family members' willing and their economic burden and doctors' assessment, also influenced the final decision of the management strategy. The cost of CTO-PCI was at least thirty to fifty thousand yuan (nearly 4.3 to 7.2 thousand dollars) in our hospital and was relatively high for some families. The decision to perform CTO-PCI was at the discretion of the interventional cardiologists and the patients and their family members'. The baseline clinical and procedural characteristics were collected from the dedicated database and medical records. Clinical end points were obtained from clinical hospital records, visits, or telephone contacts with living patients or family members. The institutional review board approved the present study.

### 2.2. Treatment Strategy

MT comprised the use of antiplatelet medication, aggressive lipid-lowering therapy, blockade of the rennin-angiotensin system, *β*-blockers, and nitrates. Coronary interventions were performed according to standard techniques. Beginning at least 24 hours before the procedure, all patients were prescribed a loading dose of aspirin (300 mg) and/or clopidogrel (300 mg) before PCI. For patients with more than one CTO, only one CTO vessel was targeted and no further attempt was made during the study period. After the procedure, a dual antiplatelet therapy with aspirin (100 mg/day) and clopidogrel (75 mg/day) was prescribed at least 12 months. All patients underwent two-dimensional echocardiography. In presence of normal wall motion in the territory supplied by the CTO artery, no further viability testing was performed.

### 2.3. Definitions and Study Outcomes

A “CTO lesion” was defined as an obstruction of a native coronary artery with a thrombolysis in myocardial infarction (TIMI) flow grade of 0 on angiography and estimated duration of >3 months [5]. A successful PCI was defined as a final residual stenosis <20%, with a TIMI grade flow ≥2 after stent implantation. The primary endpoint was the incidence of cardiac death during follow-up after PCI. The secondary endpoint was major adverse cardiac event (MACE), defined as the composite of cardiac death, myocardial infarction (MI), and target vessel revascularization (TVR). Cardiac death was defined as a death due to cardiovascular cause in absence of established cardiovascular etiology. MI was defined as an elevation of creatine kinase-MB fraction or troponin-T/troponin-I greater than the upper limit of normal with concomitant ischemic symptoms or electrocardiographic findings indicative of ischemia. TVR was defined as repeat revascularization of a CTO vessel [18, 19].

### 2.4. Statistical Analysis

Data are presented as percentages and mean ± standard or median (IQR) as appropriate. Categorical data were tested with the chi-square test or Fisher's exact test. Continuous variables were compared using the Student's *t*-test or Mann–Whitney *U* test. Event-free survival was calculated using the Kaplan–Meier method and compared with the log-rank test. Cox proportional hazards methods were used to estimate the independent effect of multiple independent variables on the risk of cardiac death. Univariate variables with *p* values < 0.05 were included in the multivariate model. All tests were two-tailed. A *p* value of <0.05 was considered significant. SPSS version 24 software (IBM, New York, USA) was used for statistical analysis.

## 3. Results

### 3.1. Baseline Characteristics

After exclusion, a total of 1294 patients with 1520 CTOs were enrolled in this study. The female group included 304 (23.5%) patients, and the male group included 990 (76.5%) patients. In the female group, they were divided into two groups: 177 in the MT group and 127 patients in the successful PCI group. In the male group, they were divided into two groups: 623 in the MT group and 367 patients in the successful PCI group (Figure 1).

Table 1 shows the baseline, angiographic, and procedural characteristics and in-hospital outcome of the enrolled patients. Compared to male patients, female patients were older and had more frequently hypertension, diabetes mellitus, dyslipidemia, and chronic kidney disease (CKD) and were less likely to have smokers, previous MI, and previous PCI. Women presented with less lesion bending (>45°) and other angiographic and procedural characteristics were similar between the two groups. As for procedural complications and in-hospital outcomes, there were no significant differences in the prevalence of coronary dissection, coronary perforation, and in-hospital death.

Table 2 shows the baseline clinical, angiographic, and procedural characteristics of female and male patients in the medical therapy and successful PCI groups. In the female group, patients in the MT group were older and more often had previous MI, CKD, taking clopidogrel. Branched CTO, CTO of left circumflex coronary artery (LCX), blunt stump, high J-CTO score, and SYNTAX score were presented more frequently in patients in the MT group compared with patients in the successful PCI group. In the male group, as compared with patients referred for successful PCI, those referred for MT were older and more likely to have previous MI, CKD, heart failure, branched CTO, CTO of LCX, blunt stump, and calcification, with high J-CTO score and SYNTAX score, but low left ventricular ejection fraction (LVEF), and were less likely to have CTO of the left ascending coronary artery.

### 3.2. Clinical Outcomes

**Figure 2:**
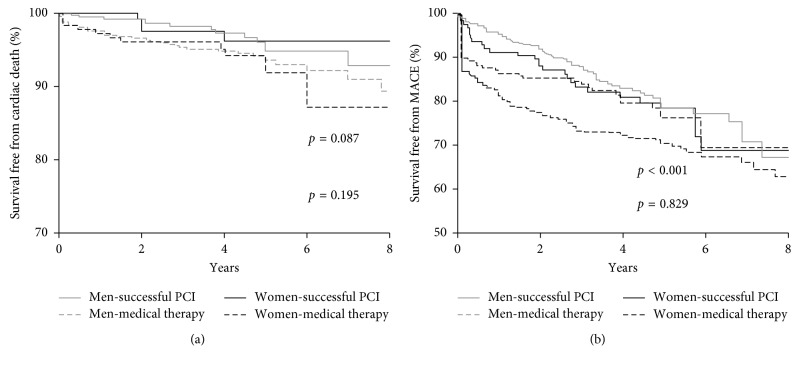
Kaplan–Meier curves for cardiac death (a) and MACE (b) during follow-up for successful CTO-PCI versus medical therapy in male and female patients. CTO, chronic total occlusion; MACE, major adverse cardiovascular events; PCI, percutaneous coronary intervention.

The median overall follow-up duration was 3.6 (IQR, 2.1–5.0) years. In the female group, no significant differences were observed between the MT and successful CTO-PCI groups in terms of cardiac death (MT vs. successful PCI: 6.8% vs. 3.9%, *p*=0.287) and MACE (20.9% vs. 21.3%, *p*=0.810). In the male group, the occurrence of cardiac death (MT vs. successful PCI: 6.6% vs. 3.8%, *p*=0.066) was comparable between the two groups. The MACE rate (30.0% vs. 18.5%, *p* < 0.001) was significantly higher in MT group (Table 3) (Figure 2).

**Figure 3:**
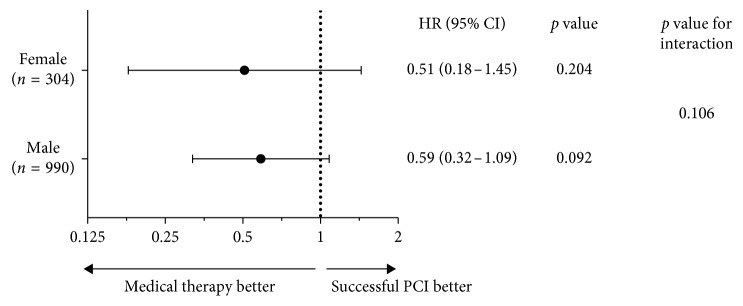
Sex subgroup analysis for cardiovascular mortality. CI, confidence interval(s); HR, hazard ratio; PCI, percutaneous coronary intervention.

There was no significant interaction between gender and treatment strategy in terms of cardiovascular mortality (*p*=0.106). The cardiovascular survival benefit after successful PCI was similar in female and male patients (Figure 3).

Table 4 shows independent predictors of cardiac death in female and male patients. After multivariate analysis, heart failure (hazard ratio [HR] 3.40, 95% confidence interval [CI] 1.23–9.40, *p*=0.018) was associated with a higher cardiac death rate in female patients; age (per-year increment) (HR 1.07, 95% CI 1.04–1.11, *p* < 0.001) and calcification (HR 3.57, 95% CI 2.05–6.25, *p* < 0.001) were independent predictors of cardiac death in male patients.

## 4. Discussion

We compared the long-term clinical outcomes of medical therapy versus successful CTO-PCI in female and male patients with CTOs. The main findings of our study are as follows: (1) only 23.5% of the patients with CTOs were female; (2) female patients were significantly older and had more frequent hypertension, diabetes mellitus, dyslipidemia, and CKD; (3) successful CTO-PCI was not associated with a reduced risk of cardiovascular mortality or MACE as compared with MT alone in female patients with CTOs; (4) successful CTO-PCI was associated with a lower rate of MACE as compared with MT alone in male patients with CTOs. To the best of our knowledge, this is one of the largest studies to compare the impact of gender on long-term clinical outcomes associated with successful CTO-PCI versus MT in unselected CTO patients.

Only a small minority of patients (23.5%) in the current study were female which was consistent with previous studies [13, 15]. In this high-risk patient cohort with advanced CAD, female patients were on average older than male patients when they first undergo invasive cardiovascular procedures, presumably due to the potentiating protective effects of oestrogen against coronary atherosclerosis until menopause, so the CAD process may be delayed. Furthermore, female patients were more frequently presented with hypertension, diabetes mellitus, dyslipidemia, and CKD which increase the risks associated with PCI, and these multiple comorbidities probably explain the low percentage of women recorded in our study as well as in other studies [7, 16, 19, 20]. In addition, female patients have more intraoperative and postoperative complications, including coronary perforation, bleeding, and contrast-induced nephropathy [15, 21]. Therefore, some interventional cardiologists were less often to perform CTO-PCI in female patients [16].

According to the Clinical Outcomes Utilizing Revascularization and Aggressive Drug Evaluation (COURAGE) trial, which conducted in patients with stable CAD, PCI was not associated with reducing the risk of death or other MACE when added to optimal medical therapy [22]. Similarly, our study also suggested that successful CTO-PCI did not reduce the prevalence of cardiac death in patients with CTOs probably because a large part of our cohort had stable coronary disease, and a similar study population was also included in the COURAGE trial.

Several studies had compared the clinical outcomes of successful CTO-PCI with failed PCI among female patients with CTOs, and had mainly shown a better outcome with regard to successful PCI [13, 23]. In our study, patients in the MT group did not undergo a CTO-PCI attempt, a population that has not been considered previously [17, 18]. Accordingly, in contrast to previous studies, our study better reflects the overall risk and clinical significance of PCI compared with medical therapy alone in patients with coronary CTOs [18].

Until now, due to the relative paucity of literature regarding sex-related differences in CTO outcomes, clinical outcome of CTO-PCI in this population is unknown. In the present study, we found that successful PCI is not associated with reduced prevalence of cardiac death, as compared with MT alone among female and male patients with CTOs, consistent with the finding of previous studies [1, 2]. Our previous study also suggested that successful CTO-PCI did not reduce cardiac death or MACE compared with MT [11, 24]. Furthermore, there was also no significant reduction in MACE rate after successful CTO-PCI in female patients. Those female patients who had medical therapy alone tended to be older and more often had CKD, CTO of left LCX, blunt stump, high J-CTO score, and SYNTAX score and were also unsuitable for PCI. These findings suggest that, considering multiple comorbidities, the high prevalence of intraoperative and postoperative complications and prognosis, as well as high expense, aggressive CTO-PCI should be considered carefully in female patients with coronary CTOs.

Interestingly, the present study showed only a reduced MACE after successful CTO-PCI in male patients, which was in accordance with previous one [25]. One possible explanation may be the relatively small sample size of the female cohort in this study. Moreover, a previous study from the multinational CTO registry and meta-analysis had turned out that gender was not independently associated with adverse outcomes [26, 27].

Several limitations should be taken into consideration. First, this was not a randomized trial and selection bias could have occurred. Second, a relatively low number of women were included. Third, the amount of viable myocardium was not routinely evaluated, which may affect the results. The expense of the test was relatively high for most families, and many CTO patients refused to accept the test, even after doctors' explanation. However, all patients in this study underwent two-dimensional echocardiography which was relatively cheap and acceptable for most patients and we used it to evaluate LV function. In presence of normal wall motion in the territory subtended by the CTO artery, no further viability testing was performed. Fourth, since this was a retrospective cohort study, routine collection of postprocedural cardiac enzymes was not performed in every patient from the study beginning, only in the case that patients suffered intraoperative myocardial or vascular injury or were present with sustained angina after operation. However, postoperative electrocardiogram was performed in every patient. Randomized controlled trials are needed to investigate clinical outcomes of medical therapy and successful percutaneous coronary intervention in both female and male patients.

## 5. Conclusions

A minority of CTO patients (23.5%) were women. Successful CTO-PCI was not associated with reduced the risk of cardiac death compared with medical therapy alone in both female and male patients. However, successful CTO-PCI reduced the rate of MACE in male patients. Aggressive CTO-PCI should be considered carefully among female patients. Larger randomized controlled trials are needed to support these findings.

## Figures and Tables

**Table 1:** Baseline clinical, angiographic, and procedural characteristics and in-hospital outcome in female and male patients with CTOs.

	Female (*n* = 304)	Male (*n* = 990)	*p* value
Age, years	68.3 ± 8.5	62.8 ± 10.5	<0.001
Smoking (%)	21 (6.9)	506 (51.1)	<0.001
Hypertension (%)	246 (80.9)	634 (64.0)	<0.001
Diabetes mellitus (%)	145 (47.7)	319 (32.2)	<0.001
Dyslipidemia (%)	235 (77.3)	693 (70.0)	0.008
Familial history of CAD (%)	31 (10.2)	118 (11.9)	0.238
Previous MI (%)	120 (39.5)	481 (48.6)	0.003
Previous PCI (%)	34 (11.2)	157 (15.9)	0.024
CKD (%)	40 (13.2)	69 (7.0)	0.001
Heart failure (%)	54 (17.8)	178 (18.0)	0.914
LVEF (%)	53.5 ± 8.5	52.5 ± 9.3	0.121
Baseline medication			
Aspirin (%)	299 (98.4)	970 (98.0)	0.677
Clopidogrel (%)	280 (92.1)	920 (92.9)	0.628
Statin (%)	287 (94.4)	945 (95.5)	0.455
*β* blocker (%)	235 (77.3)	757 (76.5)	0.763
ACEI or ARB (%)	203 (66.8)	627 (63.3)	0.274
One CTO lesion (%)	268 (88.2)	840 (84.8)	0.150
Two CTO lesions (%)	33 (10.9)	140 (14.1)	0.141
LAD (%)	101 (33.2)	358 (36.2)	0.349
LCX (%)	86 (28.3)	278 (28.1)	0.944
RCA (%)	143 (47.0)	482 (48.7)	0.615
Multivessel disease (%)	246 (80.9)	808 (81.6)	0.710
Proximal or mid			
CTO location (%)	225 (74.0)	755 (76.3)	0.424
Branched CTO	45 (14.8)	145 (14.6)	0.946
Blunt stump (%)	134 (44.1)	465 (47.0)	0.377
Calcification (%)	69 (22.7)	188 (19.0)	0.156
Bending >45° (%)	121 (39.8)	465 (47.0)	0.028
Length ≥20 mm (%)	175 (57.6)	629 (63.5)	0.061
J-CTO score	1.59 ± 1.24	1.74 ± 1.12	0.162
SYNTAX score	20.1 ± 7.6	22.5 ± 8.7	0.073
Contrast volume (ml)	177 ± 81	179 ± 88	0.977
Number of stents	1.34 ± 0.68	1.37 ± 0.70	0.995
Total stent length (mm)	38.1 ± 21.3	37.7 ± 22.6	0.758
Coronary dissection (%)	0	2 (0.2)	0.999
Coronary perforation (%)	1 (0.3)	1 (0.1)	0.999
In-hospital death (%)	1 (0.3)	6 (0.6)	0.999

Values are presented as the mean ± standard deviation or *n* (%). ACEI, angiotensin-converting enzyme inhibitor; ARB, angiotensin-receptor blocker; CAD, coronary artery disease; CKD, chronic kidney disease; CTO, chronic total occlusion; J-CTO, Japanese-chronic total occlusion; LAD, left ascending coronary artery; LCX, left circumflex coronary artery; LVEF, left ventricular ejection fraction; MI, myocardial infarction; PCI, percutaneous coronary intervention; RCA, right coronary artery.

**Table 2:** Baseline clinical, angiographic, and procedural characteristics of female and male patients in the medical therapy and successful PCI groups.

	Female			Male		
	Medical therapy (*n* = 177)	Successful PCI (*n* = 127)	*p* value	Medical therapy (*n* = 623)	Successful PCI (*n* = 367)	*p* value
Age, years	69.6 ± 8.5	66.6 ± 8.2	0.001	63.5 ± 10.8	61.8 ± 9.9	0.014
Smoking (%)	11 (6.2)	10 (7.9)	0.574	315 (50.6)	191 (52.0)	0.652
Hypertension (%)	140 (79.1)	106 (83.5)	0.339	408 (65.5)	226 (61.6)	0.216
Diabetes mellitus (%)	91 (51.4)	54 (42.5)	0.126	199 (31.9)	120 (32.7)	0.806
Dyslipidemia (%)	140 (79.1)	95 (74.8)	0.407	437 (70.1)	256 (69.8)	0.901
Familial history of CAD (%)	15 (8.5)	16 (12.6)	0.241	78 (12.5)	40 (10.9)	0.447
Previous MI (%)	80 (45.2)	40 (31.5)	0.016	326 (52.3)	155 (42.2)	0.002
Previous PCI (%)	20 (11.3)	14 (11.0)	0.940	96 (15.4)	61 (16.6)	0.637
CKD (%)	30 (16.9)	10 (7.9)	0.022	53 (8.5)	16 (4.4)	0.011
Heart failure (%)	37 (20.9)	17 (13.4)	0.091	133 (21.3)	45 (12.3)	<0.001
LVEF (%)	52.7 ± 9.3	54.7 ± 7.2	0.338	51.4 ± 9.6	54.3 ± 8.3	<0.001
Baseline medication						
Aspirin (%)	174 (98.3)	125 (98.4)	0.935	609 (97.8)	361 (98.4)	0.508
Clopidogrel (%)	158 (89.3)	122 (96.1)	0.030	572 (91.8)	348 (94.8)	0.074
Statin (%)	167 (94.4)	120 (94.5)	0.959	591 (94.9)	354 (96.5)	0.245
*β* blocker (%)	132 (74.6)	103 (81.1)	0.180	480 (77.0)	277 (75.5)	0.574
ACEI or ARB (%)	118 (66.7)	85 (66.9)	0.962	405 (65.0)	222 (60.5)	0.154
One CTO lesion (%)	157 (88.7)	111 (87.4)	0.730	528 (84.8)	312 (85.0)	0.911
Two CTO lesions (%)	18 (10.2)	15 (11.8)	0.650	88 (14.1)	52 (14.2)	0.985
LAD (%)	56 (31.6)	45 (35.4)	0.488	207 (33.2)	151 (41.1)	0.012
LCX (%)	58 (32.8)	28 (22.0)	0.041	198 (31.8)	80 (21.8)	0.001
RCA (%)	81 (45.8)	62 (48.8)	0.599	309 (49.6)	173 (47.1)	0.455
Multivessel disease (%)	141 (79.7)	105 (82.7)	0.509	511 (82.0)	297 (80.9)	0.667
Proximal or mid						
CTO location (%)	126 (71.2)	99 (78.0)	0.185	482 (77.4)	373 (74.4)	0.287
Branched CTO	33 (18.6)	12 (9.5)	0.026	104 (16.7)	41 (11.2)	0.018
Blunt stump (%)	95 (53.7)	39 (30.7)	<0.001	333 (53.5)	132 (36.0)	<0.001
Calcification (%)	46 (26.0)	23 (18.1)	0.106	144 (23.1)	44 (12.0)	<0.001
Bending >45° (%)	72 (40.7)	49 (38.6)	0.713	296 (47.5)	169 (46.0)	0.656
Length ≥20 mm (%)	108 (61.0)	67 (52.8)	0.151	401 (64.4)	228 (62.1)	0.479
J-CTO score	1.75 ± 1.29	1.37 ± 1.13	0.017	1.87 ± 1.21	1.52 ± 1.06	<0.001
SYNTAX score	21.7 ± 7.2	18.4 ± 7.8	0.046	23.7 ± 9.3	20.1 ± 6.9	0.003
Contrast volume (ml)	144 ± 67	222 ± 77	<0.001	148 ± 73	230 ± 87	<0.001
Number of stents	0	1.34 ± 0.68	—	0	1.37 ± 0.77	—
Total stent length (mm)	0	38.1 ± 21.3	—	0	37.7 ± 22.6	—

Values are presented as the mean ± standard deviation or *n* (%). ACEI, angiotensin-converting enzyme inhibitor; ARB, angiotensin-receptor blocker; CAD, coronary artery disease; CKD, chronic kidney disease; CTO, chronic total occlusion; J-CTO, Japanese-chronic total occlusion; LAD, left ascending coronary artery; LCX, left circumflex coronary artery; LVEF, left ventricular ejection fraction; MI, myocardial infarction; PCI, percutaneous coronary intervention; RCA, right coronary artery.

**Table 3:** Clinical outcomes in female and male patients during follow-up.

	Female			Male		
	Medical therapy (*n* = 177)	Successful PCI (*n* = 127)	*p* value	Medical therapy (*n* = 623)	Successful PCI (*n* = 367)	*p* value
Cardiac death (%)	12 (6.8)	5 (3.9)	0.287	41 (6.6)	14 (3.8)	0.066
MI (%)	13 (7.3)	8 (6.3)	0.723	52 (8.3)	22 (6.0)	0.174
TVR (%)	17 (9.6)	16 (12.6)	0.408	119 (19.1)	49 (13.4)	0.020
MACE (%)	37 (20.9)	28 (21.3)	0.810	187 (30.0)	68 (18.5)	<0.001

Values are presented as *n* (%). MACE, major adverse cardiovascular events; MI, myocardial infarction; PCI, percutaneous coronary intervention; TVR, target vessel revascularization.

**Table 4:** Multivariable predictors of cardiac death in female and male patients.

	HR (95% CI)	*p* value
Female		
Heart failure	3.40 (1.23–9.40)	0.018
CKD	2.10 (0.69–6.39)	0.190
Male		
Age (per-year increment)	1.07 (1.04–1.11)	<0.001
Calcification	3.57 (2.05–6.25)	<0.001
Heart failure	1.58 (0.86–2.91)	0.139
CKD	1.68 (0.83–3.41)	0.146

CI, confidence interval(s); CKD, chronic kidney disease; HR, hazard ratio.

## Data Availability

The data used to support the findings of this study are available from the corresponding author upon request.
